# Boundary crossing: an experimental study of individual perceptions toward AIGC

**DOI:** 10.3389/fpsyg.2023.1185880

**Published:** 2023-04-20

**Authors:** Wei Tao, Shuang Gao, Yilang Yuan

**Affiliations:** School of Journalism and Communication, Tsinghua University, Beijing, China

**Keywords:** AIGC, AI-mediated communication, anthropomorphism, autonomy, psychological dynamics

## Abstract

Artificial Intelligence (AI) Generated Content has made great progress in many fields. Those AI art works gradually reshape contemporary understanding of creativity. The unique creative ability of human beings may also be challenged. This paper takes AIGC as the research object and carries out a grouping experiment based on 240 participants. We found that Anthropomorphism and Autonomy have no effect on the evaluation of AI paintings and AI “painter” identity, but in together their have a combined positive impact on both independent variables. The existence of moderating effect reveals the phenomenon of on-the-spot stimulation similar to the strong effect theory. Meanwhile, the evaluation of paintings positively affects the perception of AI “painter” identity. The subjectivity of AI comes from the double superposition of its external and inner characteristics, which may suggest AI with both human-like appearance and function can be regarded as a person with social role identity.

## Introduction

Artificial Intelligence Generate Content (AIGC) has been applied in the production and life of human society. Chatgpt reached 1 million users within a week of its launch, and AI paintings reached a bidding price of 1.1 million yuan at an auction. Contemplating AI creativity can help us to look beyond the economic paradigm and consider key traits of human creativity and the creation process, some aspects of which are successfully emulated by AI (Lee, 2022: 601). Technology is used to acquaint people with art by sharing it over the Internet. Moreover, since it was found that technology may increase the experience that the arts provide, it is being adopted by museums and art galleries (Modliński, 2019). However, how to view the creativity of artificial intelligence, understand the social role that artificial intelligence may have, etc., are all questions that still need to be answered urgently. The generation of artificial intelligence has already reflected the intelligence of creation, generating unexpected effects under the framework of the algorithm. But whether to accept the artistry and autonomy of artificial intelligence-generated content is actually a cognitive issue (Gu and Wang, 2021: 102), and it needs to be tested in actual scenarios. There is also a lack of empirical research on public perception of the creativity of intelligent machines (Hong et al., 2020a: 1930).

The premise of cognitive research on the creativity of intelligent machines is to regard machines as subjects with “creative ability” that are human-like or even surpass human beings. The discourse of creative industries is a de-humanizing creativity (Lee, 2022: 601). But whether it is a process of reducing creativity and losing subjectivity remains to be studied. Previous research has focused on evaluating machine-like performance of artificial intelligence, such as the logical process of news writing by machine (Spence et al., 2019; Wu and Wen, 2021: 134). In these studies, artificial intelligence is not expected to have room for active play, and people evaluate whether their performance has achieved the intended effect. The conclusion of the research can only repeatedly prove the “instrumental nature” of intelligent machines, ignoring the possible creativity of artificial intelligence under the condition of technological progress, because creation first means exceeding expectations. There are two reasons for the above-mentioned research limitations. One is that under the classic perspective of the human-machine relationship, machines as production tools do not have any subjectivity. The second is that under the limitation of technology, the functions of machines can realize are relatively rudimentary, and it is impossible to imagine that machines have autonomous behavior or even consciousness within the scope of human cognition.

Past research shows that humans perceive the value of paintings made by AI as lower than the value of those made by humans when the creator of the work is known (Fortuna and Modliński, 2021: 201). In certain scenarios, the perceptual quality of intelligently created paintings may be better. People value cyborg’s artwork similarly to human-generated artwork when contextual cue is human (Fortuna et al., 2022). However, this conclusion is still relatively vague. One of the methods is to regard the content as a social interaction involving communication and dissemination, and speculate whether artificial intelligence can be a social participant (Hertzmann, 2018:18), this view can be generalized into the theory of Computers Are Social Actors (CASA, theory of Computers Are Social Actors). The second is to discuss the role and identity of artificial intelligence with human beings as the subject. Experiments have shown that it is difficult for people to directly distinguish whether the content is generated by artificial intelligence or created by humans without telling them the identity of the creator of the content (Elgammal et al., 2017: 20). After the identity of the creator, people’s evaluation of the content will be different due to the different attributes of the creator. It is believed that it is difficult for artificial intelligence to form a unique personal style (Hong and Curran, 2019: 1932). This kind of view is a kind of presupposition that people have about the creation of artificial intelligence. After the program is written and the instruction input is completed, the process of artificial intelligence generating content is invisible. This invisibility is the space for artificial intelligence to play independently. And it is a technological self-driven process that is difficult for people to control. Therefore, aiming at the subdivided field of artificial intelligence painting, this study first defines the characteristics of AIGC’s anthropomorphism and creative autonomy through experiments, and then explores the influencing factors of the value and role of individual cognition of artificial intelligence.

In this context, this paper offers two contributions. The first contribution is methodological, as it defines the creativity and external performance of AI painters for the first time, and examines the audience’s perception of AI-created paintings in specific contexts through these two dimensions. This attempt supplements the discussions by Lee, Fortuna, Modliński, and others, and accurately demonstrates the magnitude of this impact. The second contribution is theoretical, data suggesting that AI can only be considered as a new social identity feature that could replace humans if it meets both external appearance and internal functional requirements. Most of the time, limited creativity causes AI to still be viewed unfavorably by people. Human perspectives on intelligent machines are still influenced by functionalism. The process of embodiment and autonomy in intelligent machines is fascinating; however, the decisive factor ultimately lies in human perception of their core values, which also affects subsequent willingness to use them.

## Literature review

### Technology and topic evolution of AIGC

Artificial intelligence painting has gone through multiple stages of technological advancement. In 2014, Goodfellow proposed the algorithm model of GAN (Generative adversarial networks), which can generate high-definition fuzzy images into high-definition images with rich details (Wang et al., 2017: 326), can also perform painting style transfer, which can be applied to the generation of paintings of different styles (Hung et al., 2020: 2). In 2015, based on the convolutional neural network, the computer vision program DeepDream simulated the human brain to conduct hierarchical visual analysis of complex images: through the extraction, recognition and combination of layer by layer images, finally generate images (Arthur, 2019: 78). During the same period, Gatys, and others proposed the Neural Style method, which alleviated the problems of image distortion and content loss, and then the Fast Neural Style method accelerated the operation speed of style transfer (Li and Wan, 2020: 177). In 2021, OPENAI successively launched DALL⋅E and CLIP, two neural networks linking text and images, which brought an upsurge of people at home and abroad using AI to paint.

Character classification, painting effect, copyright ownership, creation ethics, etc., are the frontier issues of artificial intelligence painting. Previous studies have found that people’s low artist identity for artificial intelligence is due to the lack of emotional motivation and insufficient degree of autonomy (Tigre Moura and Maw, 2021: 137). But if we compare the cognitive behavior of human beings and use “S (stimulus)-O (object)-R (response)” to correspond to “input-black box-output,” then artificial intelligence can also establish its own cognition (Gu and Wang, 2021:102). The evaluation of art also affects the acceptance and recognition of artificial intelligence-generated art. Painting itself is faced with the problem of dealing with the relationship between real things and artistic creation. AI painting provides ways such as style transfer and defamiliarization of everyday things, providing experimental tools and rich cases for this relationship processing. On the issue of copyright and ethics, on the one hand, the originality of artificial intelligence-generated objects is affirmed by people, but the protection of artificial intelligence-generated objects is still in essence a respect for the fruits of human labor (Shi, 2018: 144). At present, artificial intelligence is still in the stage of algorithmic intelligence, and it is not suitable to become the subject of rights (Wang and Fu, 2017: 36). On the other hand, AI painting can generate new images by changing the original images, which has potential infringement risks. However, some creators marked the function formula of the GAN model as a signature in the lower right corner of the painting, and regarded AI as the author, as one of the methods to avoid copyright disputes.

### Artificial intelligence as a “Dasein” subject

Artificial intelligence has only acquired its subjectivity in recent years, and the shift in research has only just begun. On the one hand, artificial intelligence assists humans in decision-making, such as assisting designers in product design (Lai, 2022: 2). The organon of the industrial age believes that machines are tools that replace humans to do part of the labor and are extensions of humans (Chen, 2017: 40). This kind of evaluation itself regards the machine as a subordinate tool of human beings, and the measurement of the effectiveness of the tool ignores the important dimension of machine autonomy. With the development of artificial intelligence technology, computer applications are more and more deeply involved in human daily life, and people’s way of thinking and living habits are virtually shaped by computer software (Bie, 2019: 32). Intelligent machines gradually get rid of human intervention in the content creation process, and gradually transform from imitating human thinking and behavior to equal or even surpass human creativity.

In the social media information model (MAIN), modality refers to the multi-modal presentation of information content, such as text, audio, video, etc. Different information modalities leave different decoding spaces for users, and also bring different levels of trust. Agency means that users can judge the source and credibility of information through different clues (Sundar, 2008: 83). The different elements contained in the information determine how much users like it, and other users’ feedback on similar information also affects people’ s evaluation of the information (Men and Muralidharan, 2017: 81). Interactivity is the degree of user involvement with information content and interactive interface (Sundar, 2008: 85). If the machine can accurately match the user’s needs in different situations and meet the user’s expectations, it will deepen the relationship between man and machine and change people’s psychological presuppositions toward machines. The purpose of this study is to find the important factors affecting the understanding of the creativity of artificial intelligence drawing. Among them, the “thinking” and “intelligence” of computers are metaphors of language and thinking (Chen, 2017: 42). When the output of the machine corresponds to the input of the public, it will enhance the credibility of the public’s perceived information. When the information clues are more detailed and complete, it will also enhance the public’s favorable impression of the information content (Zhou and Wang, 2022: 64). As a result, the overall perception of the appearance of artificial intelligence, the quality of details of the produced content, etc., may interfere with the public’s recognition of artificial intelligence creation. At the same time, the judgment of the work comes from the interaction between the mind, the brain, and the mind and the brain. Therefore, it is necessary to include people’s impressions of AIGC and people’s real reactions after seeing AIGC into the scope of research.

### The cognitive double helix: anthropomorphism and autonomy

Modern collaborative methods in society have gradually become AI-assisted, just as Morgan predicted 30 years ago, the system will tie people down to the machine more and more (Morgan, 1989: 61). In the Industry 4.0 era, it is growing in prominence a trend called techno-empowerment which is defined as giving autonomy in decision-making to intelligent technology (Modliński and Gladden, 2022: 373). But the transfer of autonomy to machines is subject to certain conditions. Similar to the use and gratification theory and the technology acceptance model, existing research indicates that more positive attitudes and higher trust, perceived usefulness, and perceived ease of use are correlated with higher intention to allow the autonomous assistant independence in decision-making (Modliński, 2022: 13). Moreover, the more human-like a non-human agent is, the higher the intention to empower it—but only if this agent simultaneously provides functional and visual anthropomorphic cues explainable by the mimicry effect (Modliński, 2022: 15).

However, as the uncanny valley effect suggests, the degree of physical embodiment might, after reaching a certain point, evoke fear and resistance in people (Mori et al., 2012: 98). Therefore, from the perspective of techno-empowerment, A meticulous plan is crucial for the embodiment of machine, since the shift from “disembodied” to “embodied” could change the cognitive processes individuals rely on when evaluating technology. Concurrently, acquiring a tangible form may significantly impact human-machine interactions, encompassing the approval of techno-empowerment by humans—particularly when the form presents anthropomorphic indicators. It is worth to show the inner connections between techno-empowerment and perceptions on automatic arts produced by machine.

Based on the above discussion, we can identify two key elements: one is the functional aspect, which measures the intelligence and autonomy of machines; the other is the external representation, exploring the connection between machine and human appearances, also known as “embodiment.” In Modliński’s research, the core focus is on whether and under what circumstances people are more inclined to empower machines. This study goes further, examining people’s attitudes and tendencies in situations where machines have already been highly techno-empowered, to measure the impact of such techno-empowerment and anthropomorphic appearances on people’s cognition.

Therefore, we uses Anthropomorphism and Autonomy to measure the process of public perception and identification with AI creations. Broadly speaking, anthropomorphism is often defined as resembling human-like sensory, mental states, and behavioral characteristics (Epley et al., 2007: 865; Airenti, 2015: 118). The ontological object of anthropomorphism is human beings, but it is not unique to human beings. Emphasizing that anthropomorphism is thus simply a comparison of the referent to certain characteristics of humans is a manifestation of similarity, representing a specific human-like interpretation of existing physical characteristics and behaviors (Epley et al., 2008: 144). Even if the referent lacks any evolutionary connection with humans, even if it is different from humans in material composition, it does not indicate any superiority or inferiority evaluation, and it does not depend on the ontological status of humanoid entities.

Currently, public perceptions and attitudes toward anthropomorphism of AI, and some of the ethical implications of the anthropomorphism of AI (particularly in the context of social robots interacting with humans), have been discussed in numerous literatures. For example, as people interact with robots more frequently, their conceptions of robots will become more anthropomorphic (Fussell et al., 2008: 145). Studies have confirmed the impact of the anthropomorphic features of machines on human-machine communication. Study participants viewed robots and electronic media as living beings and preferred to interact with robots that had personality traits that complemented their own (Lee et al., 2006: 1462). Anthropomorphism was also related to the effectiveness of human interaction, as respondents visibly hesitated when they tried to shut down a humanoid robot (Horstmann et al., 2018: 1). Furthermore, humans tend to apply gender stereotypes to concrete robots with a humanoid appearance (Eyssel and Hegel, 2012: 2214).

In recent years, highly anthropomorphic artificial intelligence has exerted its powerful functions in various virtual and real scenes, and artificial intelligence painting is one of the fields. The purpose of humans endowing artificial intelligence with anthropomorphism features is to use familiar knowledge systems to understand unfamiliar subjects and reduce uncertainty (Epley et al., 2007: 170). There are two benefits of anthropomorphism. One is that humans can better perceive, understand and control machines; the other is that anthropomorphic artificial intelligence can better integrate into human society and strengthen the social connection between humans and machines. Researchers have traced back to the concept of anthropomorphism in European musical instruments in the 17th century, exploring the combination of anthropomorphic features and mechanical systems in music production today (Cypess and Kemper, 2018: 201). Fink found that the public is more likely to accept artificial intelligence that is highly similar to humans in terms of appearance and behavior (Fink, 2012). It can be seen that in the creation process of artificial intelligence with anthropomorphic characteristics, it fits the secular experience of “human beings as creators in a general sense” in the minds of the public, and is more in line with the existing cognitive logic of human beings. Therefore, this paper conjectures that anthropomorphic nature will also affect the public’s value judgment on the content created by artificial intelligence. Based on the above discussion, the following hypotheses are first proposed:

H1: People who experienced high anthropomorphic AI rated the painting higher than those who experienced low anthropomorphic AI.

H2: Compared with low anthropomorphic scenarios, people who experience high anthropomorphic artificial intelligence are more likely to recognize the artificial intelligence as a painter.

Autonomy is the motivation, ability, or characteristic of a behavior subject to act according to one’s own will, and is a multi-disciplinary concept. Philosopher Immanuel Kant believed that “Ought implies Can,” meaning conscious choice freedom (Chen, 2014: 69). Autonomy refers to multiple levels, such as autonomous thinking, autonomous behavior, and autonomous existence. The autonomy of artificial intelligence mainly refers to autonomous operation and creation. It should be noted that at present algorithms are still built by humans, and artificial intelligence does not yet have prior self-awareness and creative instincts (future new artificial intelligence may be created from earlier existing artificial intelligence), so the autonomy of artificial intelligence’s existence depends on humans. The autonomy discussed in this paper refers to the autonomy of creation, which means that it can operate and create content independently without any human assistance based on existing program algorithms. Currently, whether artificial intelligence has autonomy is still a matter of debate, and scholars believe that the autonomy and creativity of machines usually depend on their programmers (Elgammal, 2019: 30; Ribeiro, 2020: 380). For example, if artificial intelligence creates a painting, the public may still consider it the property of the builder of the algorithm platform. There is also research showing that the public’s perception of the creativity of artificial intelligence changes their evaluation of the content created by artificial intelligence. Compared to those who do not agree that machines have creativity, those who accept machine creativity score higher for works of art (Hong et al., 2020b: 1921). In other words, when the public regards the art created by artificial intelligence as a manifestation of its own autonomy, the public will appreciate its production more. From this, it can be seen that autonomy is not a simple dichotomous concept that can be easily judged as “yes or no,” it is a dynamic factor that can be perceived and regulated, and may affect the public’s experience of artificial intelligence creation. Based on the above discussion, this study assumes that the autonomy embodied in the process of artificial intelligence painting will affect the public’s judgment on paintings and artificial intelligence, and puts forward the following hypotheses:

H3: Paintings created by artificial intelligence with high autonomy will receive higher ratings from the public than those with low autonomy.

H4: Compared with low-autonomy scenarios, AI with high autonomy is more likely to be regarded as a painter by the public.

### Clues from social role theory and TAM theory

When artificial intelligence assists or replaces human labor, the role it plays in society should be considered. When the level of AI drawing even surpasses the limits of human beings, should the public consider it as a “real painter?” In what circumstances is AI more likely to achieve this perceptual effect? All of these thoughts surrounding AI as a subject matter need to be understood from the perspective of Role Theory in Sociology. Role theory explains an individual’s position in the social structure (Hunter, 2015: 50). As a sociological concept, it was first introduced into the field of sociology by American philosopher and social psychologist Mead (Ren, 2016: 123). In exploring the meaning of role, scholars who emphasize structure and holistic nature define the concept from the perspective of the social whole, for example, “each role has a set of rights, duties, and behavioral norms” (Fei, 1984: 63); while some scholars mainly define the role concept from the individual level: “a role is a behavior pattern associated with a particular position” (Lin, 1985: 246).

The composition of current and future social subjects will undergo structural changes due to the addition of artificial intelligence subjects. For example, the entry of various robots and virtual humans. The analysis of the social subject—whether it is an individual or an organization, needs to re-examine and clarify the nature of the subject and its social role. As an individual, artificial intelligence can be roughly divided into two categories. One is regarded as a vassal of human beings, without an autonomous social role, and as an accompaniment and derivative of human behavior. For example, if a machine cannot draw on its own, it cannot be considered a musician, but rather a software tool. The other category is based on a reliable level of intelligence, which is regarded as a social role close to human beings, and even eventually equal to human beings. Some scholars believe that there is no significant difference between humans and machines in terms of creativity, because both generate innovative ideas from old ones (Jackson, 2017: 101). However, defining autonomy (human participation) is a challenge, because the current artificial intelligence still needs human support at the bottom level. In this study, however, autonomy in the drawing process was clearly defined. If artificial intelligence can automatically generate paintings according to human painting instructions without programmer control, then it is regarded as creative autonomy. Studies have shown that artificial intelligence can compose musical compositions without external input, as long as it is trained with enough expert data. They only require the public to choose the style of music they want to listen to (Zulić, 2019: 101). The hypothesis of this study regarding the identity of the artist is based on the autonomous creation of characters, individuals can prove their social behavior (Giovagnoli, 2018: 21). The public needs to be able to perceive the creative autonomy of artificial intelligence, which leads them to accept its role as a painter. If this hypothesis is supported, independently creative AI music makers are more likely to be seen as musicians who can take responsibility for their own music than independently creative.

The social identity recognition directly comes from the evaluation of the individual’s functions. People often participate in social interaction by following role expectations (Lynch, 2007: 1469). The explanation of social identity recognition describes how expected social status and expectations determine specific social behavior patterns and focuses on performance or behavior in a given environment when defining roles (Biddle, 1986: 70). Thus, artificial intelligence itself is not a restriction on humans not becoming its painter identity. Treating artificial intelligence as a painter should focus more on the painting itself. To what extent, under what conditions, artificial intelligence can be considered a real and autonomous individual role, still needs to be explored. This paper hypothesizes that a rich-functioning, human-like artificial intelligence is more likely to be seen as valuable, thus having a certain social identity. While a functionally impaired, autonomously intelligent weak one is more likely to be considered as an accessory to humans.

**FIGURE 1:**
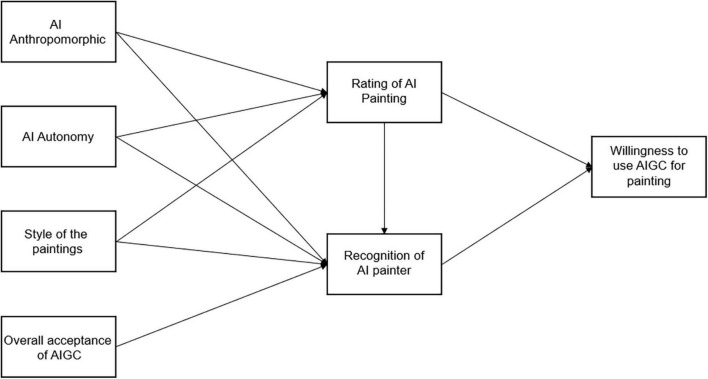
Variable analysis path model.

From the TAM theory, perceived usefulness and perceived ease of use jointly promote the adoption and use of technology (Davis, 1985). Artificial intelligence painting as a technology, therefore, further research infers that the recognition of value and identity of artificial intelligence painting may lead to the public’s acceptance and use of AIGC. For those who accept artificial intelligence painting, artificial intelligence paintings will be seen as fulfilling the responsibilities of the “painter” social role assigned to them, which will trigger a deeper level of positive attitudes toward artificial intelligence painting (model see Figure 1). This paper takes artificial intelligence painting as a starting point, by exploring the relationship between the content of artificial intelligence creation and the public’s social identity recognition, and then revealing whether this recognition will encourage the public to accept and use artificial intelligence, and puts forward the following hypothesis:

H5: The public’s ratings for artificial intelligence painting works and their recognition of the machine painter’s social identity have a positive impact on their adoption and use of artificial intelligence.

H6: Ratings on paintings and recognition of AIGC will jointly positively affect individuals’ willingness to use AIGC paintings.

## Methodology

### Experiment process

In order to test the above hypothesis, a 2 × 2 experiment was designed and conducted. The independent variables were all categorical variables, and the assigned values were high (1) and low (0). In the specific operation process, AI human-likeness (high anthropomorphism and low anthropomorphism) and AI creative autonomy (high creative autonomy and low creative autonomy) were controlled. The dependent variables of the study were the respondents’ ratings on the content of the paintings, their recognition of the identity of the AI as a painter, and their willingness to use AIGC for painting. All three dependent variables were measured as continuous variables. In addition, respondents’ overall acceptance of AIGC and the style of the paintings were covariates.

**FIGURE 2:**
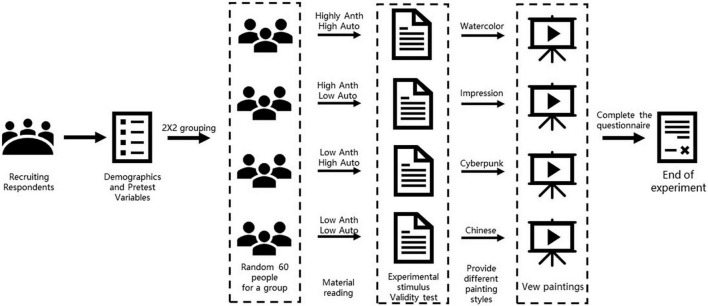
Experiment process.

In the real experiment process (see Figure 2) of college students were randomly recruit respondents through the snowball method and they were divided into four groups according to the same amount of people. Before conducting the experiment, the age of the interviewed group was first collected through an online questionnaire, and their general views and acceptance of artificial intelligence and artificial intelligence painting were asked. Subsequently, the four groups of interviewees read four corresponding articles, respectively. These articles, respectively, described four types of artificial intelligence painting platforms with different characteristics: (A) high anthropomorphism and high creative autonomy, (B) high anthropomorphism and low creative autonomy, (C) low anthropomorphism and high creative autonomy, (D) low anthropomorphism and low creative autonomy. For example, in group A, the article will describe an interactive artificial intelligence painting program with a human-like appearance, attach a sticker with a typical human form, and clearly inform the interviewee that the platform can draw independently. After the respondents finished reading the corresponding article, the researchers randomly assigned each respondent one of the four paintings and asked them to watch them carefully for 2 min. Respondents were told that the paintings they saw were created by artificial intelligence. After completing all the above steps, the interviewees were asked to rate the content of the painting they just saw, and report their views on whether artificial intelligence can become a painter, and finally, the level of willingness to use AI to paint. Choosing the college student population may have certain limitations. But millennials including college students stand out for their technology use (Pew Research Center, 2019). They are potential users of these creative tools and have a high probability of becoming the mainstream population in the future. So, they can still provide some insights. Moreover, the sample size of this group is sufficient, and the experimental process is carefully controlled, making the conclusions drawn applicable to the age range of 20−30 years old to a certain extent.

### Variable specification and measurement

The overall acceptance of AI, which refers to the individual’s existing general perception of AI before participating in the experiment, is one of the covariates measured by the questionnaire before the experiment. This variable measure was adapted from the Negative Attitudes toward Robots (NARS) Scale (Nomura et al., 2006: 28). Some words (such as paranoid) have been changed to adjectives that are more in line with the Chinese context. The study used a 5-point Likert scale consisting of 9 items. Higher scores indicate more negative attitudes toward autonomous machines (α = 0.76).

The ratings on the content of the paintings refers to the degree of satisfaction of the respondents with the paintings drawn by artificial intelligence in the experiment. This variable was adapted from the “Artificial Intelligence Composer Effectiveness Scale” (Hickey, 1999). Similar to music, painting is also a kind of art. Evaluation based on dimensions such as aesthetic appeal and creativity can intuitively show the audience’s perception of the results of artificial intelligence painting. The scale consists of 9 items, which are scored on a scale of 1−5. The higher the score, the more positive the evaluation of the quality of the painting (α = 0.90).

The degree of recognition of the identity of the AI painter refers to the degree to which the interviewees regard artificial intelligence as a real painter and have the same identity as a human being after participating in the experiment (Hong et al., 2020a: 239). The original scale was designed to test music production machines. In this paper, we have modified the items and set the questionnaire target as painting machines. It is measured by three items and scored on a scale of 1−5. The higher the score, the higher the acceptance of artificial intelligence as a painter (α = 0.89).

Artificial Intelligence humanism refers to whether the artificial intelligence that generates the painting looks like a human. This variable is measured dichotomously, with high anthropomorphism and low anthropomorphism. AI creative autonomy refers to the autonomy in the process of artificial intelligence generating paintings. This variable is measured dichotomously, with high and low creative autonomy. The willingness to use AI for painting refers to the emotional intensity of the interviewees on the subsequent use of AI after the experiment. Score on a scale of 1−5, the higher the score, the stronger the willingness to use. The style of the painting refers to the overall style of the painting. In this study, “watercolor painting,” “impression painting,” “cyberpunk,” and “Chinese painting” are selected as the four representative styles that AI can generate paintings. Distinguished by the drawing provided to the respondent in the specific questionnaire, is the covariate of the study.

## Results

### Description of basic characteristics of variables

In order to eliminate the error of the experimental operation, the research firstly aimed at two independent variables, asking the respondents “whether the level of anthropomorphism in the artificial intelligence painting in the Q1 article is high or low,” “whether the creation autonomy of the artificial intelligence painting in the Q2 article is high or low?,” and then compared between different levels using independent samples *t*-test. The results showed a significant difference between highly anthropomorphic (*M* = 3.86, SD = 1.13) and low anthropomorphic (*M* = 1.83, SD = 1.07), *t*(240) = 7.39, *p* < 0.001. There was also a significant difference between high creative autonomy (*M* = 3.41, SD = 1.05) and low creative autonomy (*M* = 1.68, SD = 1.14), t (240) = 5.13, *p* < 0.001. These results confirm the stimulating effectiveness of the material in the experiments.

The research then eliminated the experimental data whose answering time was less than or equal to 100 s, and kept the same number of people in the four groups, and finally collected 240 valid samples and the overall data can be obtained in Supplementary material. Table 1 is the basic information of the respondents. Overall, the gender ratio is relatively balanced; in terms of age, nearly 90% of the samples are 20−30 years old; as for majors, literature disciplines accounted for more than half, followed by engineering, and medical students were the least. Everyone is generally optimistic about artificial intelligence and artificial intelligence painting. Nearly 90% of the students have a total score of more than 20 points, and the highest score is 45 points; about 15% of the students are more cautious and negative.

**TABLE 1:** Basic characteristics of respondents.

Items	*n*	%
**Gender**		
Male	101	42
Female	139	58
**Age**		
Under 20	17	7
20−25	110	46
25−30	99	41
30−35	8	3
Above 35	6	3
**Major**		
Literature	176	68
Science	18	7
Engineering	54	21
Medic	10	3
**Pre-test attitude**		
Under 10	2	1
10−20	33	14
20−30	149	62
30−40	53	22
Above 40	3	1

### ANCOVA of anthropomorphism, autonomy, ratings of paintings, and AI painter identity

**FIGURE 3:**
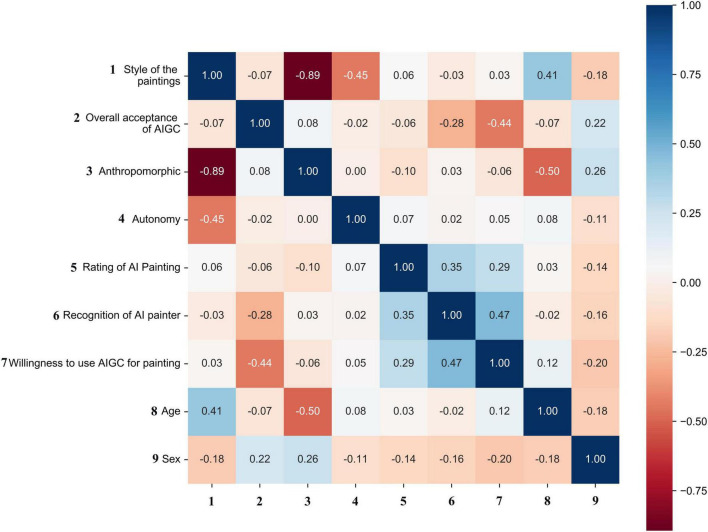
Correlation matrix.

The study first drew the correlation matrix of basic variables as a pre-observation for ANCOVA. In the Figure 3, data significance is not displayed; instead, blue (positive) and red (negative) colors are used to represent the potential numerical correlation between the two variables. it can be initially seen that there is a positive correlation between the scores given to the paintings, the degree of identification with the AI painter, and the willingness to use AIGC to paint. However, there is little correlation between humanization, autonomy and the scores given to the paintings, the degree of identification with the AI painter, and the willingness to use AIGC to paint. Instead, there are some differences in demographics.

To test H1–H4, the study conducted two sets of a two-factor analysis of covariance (ANCOVA). First, for H1 and H3, the dependent variable was the rating of painting, the independent variables were anthropomorphism and creative autonomy, and the covariates were style of painting and general perceptions of AI. Levene equivalence test indicated that ANCOVA could be conducted (*F* = 1.086, *p* = 0.356). The results in Table 2 showed that both anthropomorphism (*F* = 2.569, *p* = 0.112 > 0.05) and creative autonomy (*F* = 1.269, *p* = 0.261 > 0.05) did not have a significant effect on the ratings of paintings, while the interaction term between the two had a significant positive effect on the dependent variable (*F* = 5.388, *p* = 0.021 < 0.05). Style of painting (*F* = 3.701, *p* = 0.113 > 0.05) and general perceptions of AI (*F* = 0.963, *p* = 0.531 > 0.05) did not have a significant effect on the dependent variable. H1 and H3 were not confirmed.

**TABLE 2:** Test of the effect of anthropomorphism and creative autonomy on the rating of painting.

Items	Type III sums of squares	Degree of freedom	Mean square	F	Significance
Modified model	399.846^a^	3	133.282	3.068*	0.029
Intercept	228228.338	1	228228.338	5254.400***	0.000
Anthropomorphism	110.704	1	110.704	2.549	0.112
Creative autonomy	55.104	1	55.104	1.269	0.261
Anthropomorphism* creative autonomy	234.038	1	234.038	5.388*	0.021
Error	10250.817	236	43.436		
Total	238879.000	240			
Corrected total	10650.662	239			

*p < 0.05, ***p < 0.001; the same below. Dependent variable: rating of painting.

Another set of analysis of ANCOVA tested H2 and H4, the dependent variable was recognition of AI painter’s identity, the independent variables were anthropomorphism and creative autonomy, and the covariates were style of painting and general perceptions of AI. Levin equivalence test indicated that ANCOVA could be conducted (*F* = 0.733, *p* = 0.533 > 0.05). The results in Table 3 showed that both anthropomorphism (*F* = 0.162, *p* = 0.688 > 0.05) and creative autonomy (*F* = 0.083, *p* = 0.774 > 0.05) did not have a significant effect on the ratings of paintings, while the interaction term between the two had a significant positive effect on the dependent variable (*F* = 0.353, *p* = 0.043 < 0.05). Style of painting (*F* = 0.139, *p* = 0.937 > 0.05) and general perceptions of AI (*F* = 1.655, *p* = 0.224 > 0.05) did not have a significant effect on the dependent variable. H2 and H4 were not confirmed.

**TABLE 3:** Test of the effect of anthropomorphism and creative autonomy on the recognition of AI painter’s identity.

Items	Type III sums of squares	Degree of freedom	MS	F	Sig.
Modified model	6.779^a^	3	2.260	0.199	0.897
Intercept	20148.338	1	20148.338	1776.986	0.000
Anthropomorphism	1.838	1	1.838	0.162	0.688
Creative autonomy	0.938	1	0.938	0.083	0.774
Anthropomorphism* creative autonomy	4.004	1	4.004	0.353*	0.043
Error	2675.883	236	11.338		
Total	22831.000	240			
Corrected total	2682.663	239			

*p < 0.05; **p < 0.01; ***p < 0.001. Dependent variable: recognition of AI painter’s identity.

### Regression analysis and moderation analysis of willingness to use AIGC for painting

A Pearson correlation analysis was conducted between the “rating of painting” and the “recognition of AI painter’s identity,” a significant and positive correlation was found in Table 4 (Beta = 0.346, *p* < 0.001), H5 was confirmed.

**TABLE 4:** Correlation analysis between rating of painting and recognition of AI painter’s identity.

		Rating of painting	Recognition of AI painter’s identity
Rating of painting	Pearson correlation	1	0.346**
	Sig.(two-tailed)		0.000
Recognition of AI painter’s identity	Pearson correlation	0.346**	1
	Sig.(two-tailed)	0.000	

*p < 0.05; **p < 0.01; ***p < 0.001.

The independent variables were “rating of painting,” “recognition of AI painter’s identity,” and “rating of acceptance toward AI,” the dependent variable was “willingness to use AIGC for painting,” a multiple linear regression model was constructed (R2 = 0.35, *p* < 0.001; *F* = 42.31, *p* < 0.001). The results in Table 5 showed that there was no collinearity in the three independent variables (VIF < 5). The rating of painting significantly and positively influenced the willingness to use AIGC for painting (Beta = 0.16, *p* < 0.001); recognition of AI painter’s identity significantly and positively influenced the willingness to use AIGC for painting (Beta = 0.32, *p* < 0.001); However, there was a significant negative correlation between rating of acceptance toward AI and the willingness to use AIGC for painting (Beta = −0.35, *P* < 0.001), Because the higher the score, the more positive the attitude toward artificial intelligence, the stronger the willingness to use AIGC for painting. H6 is verified.

**TABLE 5:** Multiple regression results of willingness to use AIGC for painting.

	B	SE	Beta	t	Sig.	VIF
(Constant)	3.331	0.438		7.607	0.000	
Rating of painting	0.028	0.010	0.156	2.781	0.006	1.138
Recognition of AI painter’s identity	0.114	0.021	0.321	5.519	0.000	1.228
Rating of acceptance toward AI	−0.069	0.011	−0.347	−6.340	0.000	1.085

Dependent variable: willingness to use AIGC for painting.

The study also examined the possible moderating effect of the two independent variables between the pre-test variable ‘rating of acceptance toward AI’ and the dependent variable ‘willingness to use AIGC for painting’. Cross-tabulations XM1 (rating of painting * rating of acceptance toward AI) and XM2 (recognition of AI painter’s identity * rating of the attitude toward AI) were constructed and tested for significance, respectively. The results in Table 6 showed a moderating effect of “rating of painting” on the relationship between “rating of acceptance toward AI” and “willingness to use AIGC for painting” (*t* = 1.99 (t = 1.99, *p* = 0.48 < 0.05); “recognition of AI painter’s identity” moderated the relationship between “rating of the attitude toward AI” and “willingness to use AIGC for painting” (*t* = 2.99). There was a moderating effect of “recognition of AI painter’s identity” on the relationship between “rating of acceptance toward AI” and “willingness to use AIGC for painting” (*t* = 2.34, *p* = 0.02 < 0.05). Meanwhile, there is a certain negative correlation between the pre-experiment perception of AI and the willingness to use AI for painting. One possible explanation is that the former is a general concept, and the perception of AI also includes many other factors in daily life, such as smart homes and intelligent question-answering systems, which are not necessarily related to the use of AIGC measured in the experiment. At the same time, the former is a pre-test variable, while the latter is a variable after exposure to the experimental stimuli. However, the relationship between these two factors is worth further investigation in future research.

**TABLE 6:** Moderating effect of willingness to use AIGC for painting.

M1	Beta1	Sig.	M2	Beta2	Sig
(Constant)		0.000	(Constant)		0.000
Rating of painting	0.262	0.000	Rating of acceptance toward AI	−0.340	0.000
Rating of acceptance toward AI	−0.430	0.000	Recognition of AI painter’s identity	0.377	0.000
(Constant)		0.000	(Constant)		0.000
Rating of painting	−0.137	0.511	Rating of acceptance toward AI	−0.638	0.000
Rating of acceptance toward AI	−0.836	0.000	Recognition of AI painter’s identity	−0.092	0.659
XM1	0.564	0.048	XM2	0.496	0.020

Further analysis of the correlation coefficients between the direct and moderating effects showed that the coefficients of the independent variable “rating of acceptance toward AI” were smaller with the inclusion of the cross term (Beta1a = −0.43 > Beta1b = −0.84; Beta2a = −0.34 > Beta2b = −0.64). This suggests that individuals with the same level of acceptance toward AI in the pre-test will result in a stronger willingness to use AIGC for painting if they rating higher in the painting and recognition with the AI painter’s identity in the experiment. This means that the experiment was effective in increasing individuals’ acceptance of AIGC. The process of feeling and understanding exists after an individual is exposed to the experimental materials of AIGC, and the information observed and learned during the experiment reshapes the perception of AIGC and its applications to an individual. This experiment demonstrates that if previously uninterested in AI, individuals will change their previous attitudes and reduce negative or enhance positive attitudes after viewing the AI paintings, as a process of clinical stimulation.

## Findings and conclusion

Finally, let’s draw a conclusion. From the moment intelligent machines were born, the integration between machines and humans has never ceased. Numerous researchers have conducted studies on various aspects such as the intelligent industry, intelligent machines, and intelligent production (Lee, 2022: 609), including Lee, Modliński, Fortuna, Morgan, and Mori mentioned in this paper. Although the research methods and topics vary, the core of the repeated discussions is still about the impact of the level of machine intelligence on humans and how people view the boundary-crossing issue of “machines potentially having intelligence.” Research shows that the presence and collaboration of intelligent machines in social divisions and organizational structures are an unstoppable trend (Morgan, 1989), and human techno-empowerment of machines is reaching new heights.

However, humans have different views on the functions and appearances of intelligent machines, and such techno-empowerment is also constrained by these factors. This study, based on a specific application case of intelligent painting machines, discusses the impact of machine creative autonomy and anthropomorphism on individual cognition. This impact is distinguished from previous studies by using three dimensions: the rating of machine-generated paintings, the rating of the machine’s social role, and the willingness to use intelligent machines subsequently. These dimensions collectively reflect a person’s view of intelligent machines after being techno-empowered. Both machine autonomy and anthropomorphism are indispensable for individual perceptual effects; only when both are present can an individual have a better view of intelligent painting machines. And we also discovered that the experimental stimulation experienced on-site can alter individuals’ perceptions of AIGC. In all we call it “synergistic and powerful effect.”

Specifically, the study found that rating of the paintings significantly and positively influenced individuals’ recognition of AI painter’s identity: recognition of AI painter’s identity stemmed from an increased perception of AI’s function. There exists a process similar to a strong stimulation, where AI performance has an immediate effect on human’s perception toward it. Unlike the classical cultivation effect- where media such as television or newspapers slowly influences people’s perception - AI can change an individual’s perception of it in a short time by its generated contents.

At the same time, the subjectiveness of AI: anthropomorphism and creative autonomy have no impact on the rating of painting or the recognition of AI painter’s identity, but the products of anthropomorphism and creative autonomy have an impact on the two independent variables mentioned above, meaning that individuals do not measure a single indicator of AIGC, respectively, but rather see and assess it as a whole (with both machine and human attributes). It also implies a shift in the people’s perception of AIGC, from measuring function and effect only to taking into account of anthropomorphism and other personality attributes.

Moreover, outcomes showed moderating effects in both pairs of variable relationships. On the one hand, people were more willingly to use AIGC when they were “stimulated” by better painting content quality; On the other hand, the recognition of AI painter’s identity also have positive contribution to the use of AIGC based on initial perception of AI. This indicates that for individuals with the same initial level of AI acceptance, a higher rating of the painting and recognition of the AI painter’s identity during the experiment will lead to a stronger willingness to use AIGC for painting. This conclusion is bold and merits further validation, as it suggests that individuals’ views on AIGC may change along with the external and internal performance levels of AI, even for those who previously had some hostility toward technological empowerment and AI. After receiving experimental stimulation, there is a certain degree of change in their ideas.

Future research can be improved in several ways, such as expanding the scale of the sample group, extending the research subject from intelligent painting machines to other functions, and adopting more immersive usage experiments for the measurement process. However, the conclusions may suggest that human intelligent machines will develop toward a double helix structure of perfected autonomous inner functions and anthropomorphic external appearances. Whether it is news writing, artistic creation, or industrial production, highly intelligent machines with human-like appearances will better align with human cognitive expectations. From one perspective, the boundary between humans and machines is already blurred. Artificial intelligence with powerful technology and humanoid appearance may be able to take on a “human identity.”

## Data availability statement

The original contributions presented in this study are included in the article/Supplementary material, further inquiries can be directed to the corresponding author.

## Ethics statement

The studies involving human participants were reviewed and approved by the Journalism and Communication of Tsinghua University. The patients/participants provided their written informed consent to participate in this study.

## Author contributions

WT: conceptualization, methodology, and writing—original draft. SG and YY: writing—review and editing and software. All authors listed have made a substantial, direct, and intellectual contribution to the work, and approved it for publication.
