# Supplementary material for: Boundary crossing: an experimental study of individual perceptions toward AIGC
Source: Front Psychol. 2023 Apr 20;14:1185880. doi: 10.3389/fpsyg.2023.1185880 (PMC10159051; doi:10.3389/fpsyg.2023.1185880)
Supplement: Supplementary file 2 [file Data_Sheet_2.pdf]

Appendix (two samples of reading materials)

High Anthropomorphism & Low Autonomy

Introduction to an artificial intelligence painting

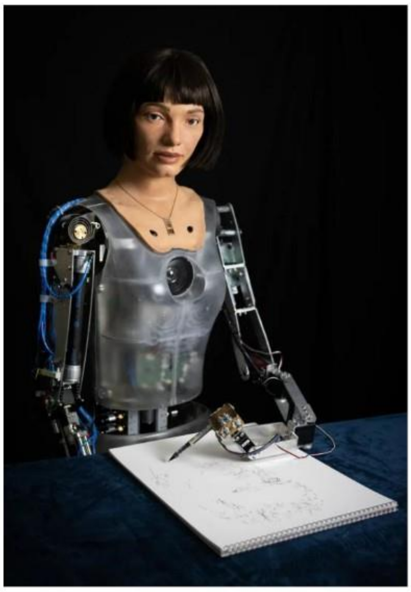

The picture above is a rendering of an artificial intelligence painting. The artificial intelligence painter Ai-Da has the same appearance as a human being, including the shape of a human body and a robotic arm capable of painting. Able to see, listen, speak, interact with the audience, and draw on the spot for the audience to see at the same time.

The unique ability of Ai-Da is that it can autonomously interact with the external environment, recognize the audience's voice and even facial expressions, collect effective information, and form a unique painting mode. As for what kind of painting Ai-Da will draw, the audience does not know, it is completely determined by Ai-Da's independent decision-making.

This artificial intelligence does not have independent comprehension and creation capabilities, and still needs the input of external human instructions to guide him to complete the painting process.

Introduction to an artificial intelligence painting

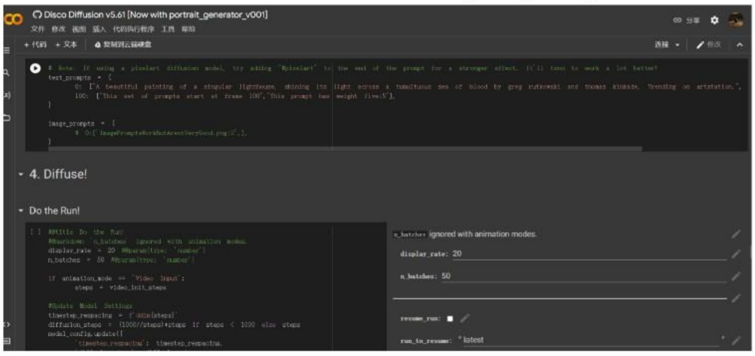

The picture above is a rendering of an artificial intelligence painting. This artificial intelligence painting platform can already complete rich painting creations, running in the background of invisible programs, and its machine learning engine is the core of creation.

However, the lack of an embodied and anthropomorphic image means that it cannot use both hands to show you face-to-face like a human being, and at the same time, it cannot interact with you physically or voice in real time. In other words, apart from the ability to create paintings, artificial intelligence itself does not have human-like appearance characteristics.

This artificial intelligence has completely independent understanding and creation capabilities, and fully performs the duties of a "painter" in the process of creating new paintings, and can paint autonomously without any instructions from the programmer. After the painting is completed, it can be displayed to the audience independently. In other words, the artificial intelligence enjoys absolute control over the painting.
